# Strategies for Reducing Fat in Mayonnaise and Their Effects on Physicochemical Properties

**DOI:** 10.3390/foods14173133

**Published:** 2025-09-08

**Authors:** Cecilia Abirached, María Noel Acuña, Tatiana Carreras, Ignacio Vieitez

**Affiliations:** 1 Food Chemistry Area, Food Science and Technology Department, Chemistry School, Universidad de la República, 2124 General Flores Ave., Montevideo 11800, Uruguay; mnoel2718@gmail.com (M.N.A.); tatianacarrerasg@gmail.com (T.C.); 2 Food Technology Area, Food Science and Technology Department, Chemistry School, Universidad de la República, 2124 General Flores Ave., Montevideo 11800, Uruguay

**Keywords:** reduced-fat mayonnaise-type sauce, physicochemical properties, oxidative stability enhancement and emulsion stability

## Abstract

Mayonnaise is a widely consumed food emulsion. Traditional mayonnaise contains approximately 70–80% lipids, making it a high-fat, calorie-dense food. This study aimed to develop a reduced-fat mayonnaise with physicochemical properties comparable to commercial low-fat formulations but with a lower oil content (<30%). Three formulations were prepared using canola oil and high-oleic sunflower oil at different concentrations (10%, 15%, and 30%), with and without the addition of synthetic antioxidants (BHA and BHT). Guar gum was used to control the viscosity of the continuous phase, adjusting its concentration between 0.75% and 1.55%. The formulations were compared with a commercial low-fat sample (MH) in terms of flow and rheological properties, color, phase separation stability, particle size, microscopy, and oxidative stability. The formulations exhibited flow behavior and Konini’s viscosity similar to MH. The 15% oil formulation (MHO-15%) had a particle size comparable to MH. Both MH and the experimental formulations exhibited a weak gel structure. To achieve the characteristic yellow color, β-carotene should be added to MHO-15%. Formulations containing canola oil and those without antioxidants showed higher susceptibility to oxidation, leading to the selection of high-oleic oil with added antioxidants. Based on these findings, a potential reduced-fat mayonnaise-type sauce could be formulated by decreasing lipid content from 30% to 15%.

## 1. Introduction

Sauces and mayonnaise are popular emulsion-based foods commonly used to improve the taste of a wide range of dishes [1]. Mayonnaise is a semi-solid oil-in-water emulsion obtained by blending oil, egg yolk, salt, vinegar, and seasonings. Conventional mayonnaise typically contains about 70–80% lipids, which makes it a food high in fat and calories [2]. The globally average mayonnaise consumption per capita varies widely, while in countries like Russia, it surpasses 5 kg per person per year, the global average is closer to 1.5–2 kg [3]. In Uruguay, the National Food Regulation (Reglamento Bromatológico Nacional, RBN) [4] stipulates that mayonnaise must contain a minimum of 65% lipids and have a maximum pH of 4.0 at 20 °C. Additionally, it defines “mayonnaise-type sauce” as a product with a minimum lipid content of 30%. The connection between diet and health is now widely acknowledged. Food scientists and mayonnaise manufacturers are increasingly developing low-fat and low-calorie versions of mayonnaise due to both health and cost considerations. Since fats have over twice the caloric density of proteins and carbohydrates, reducing fat content can significantly lower a product’s calorie count [5]. As obesity becomes an increasing concern in many markets, coordinated efforts are being made across various sectors to address the issue. This trend is pushing sauce manufacturers to adapt their product ranges in order to remain competitive. Creating healthier options requires reformulating recipes by lowering fat [6,7]. Reducing fat content while enhancing nutritional quality is also a key strategy for lowering the risk of type 2 diabetes mellitus and cardiovascular diseases [8,9]. However, fat plays a crucial role in maintaining food quality, particularly in texture, flavor, and the stability of food emulsions [9]. Several studies in the literature have focused on reducing fat in mayonnaise and sauces. Kryskova and Spivak [1] explored a strategy for enhancing mayonnaise by using different ratios of sunflower, linseed, and hemp oils with the aim of increasing the amount of polyunsaturated fatty acids, especially the Omega-3 family, and reducing lipid content. They also designed a market strategy for this modified product [1].

Carcelli et al. [8] examined a traditional full-fat mayonnaise formulation (80% oil) and three reduced-fat versions (60%, 40%, 25% oil), replacing oil with physically modified corn flour. They evaluated the physicochemical properties (storage stability, rheology, color) and sensory acceptability of both pasteurized and unpasteurized mayonnaises. Their findings indicated that physically modified corn flour is a good oil replacer in reduced-fat mayonnaise [8].

Wang et al. [10] studied a low-fat mayonnaise with a 50% oil content made with soybean oil bodies (SOBs) as an egg yolk substitute. The effects of varying ratios of xanthan gum, pectin, and modified starch as stabilizers were examined to determine their influence on the apparent viscosity, stability, texture, and microstructure of SOB-based mayonnaise. Results indicated that combining xanthan gum, pectin, and modified starch in a 2:1:1 ratio significantly improved both viscosity and stability. The research also evaluated the product’s storage stability. Compared with traditional full-egg-yolk mayonnaise, the SOB-based version exhibited superior oxidative stability and antibacterial properties, which are beneficial for shelf life.

However, fat reduction often compromises texture, stability, and mouthfeel, all of which are key sensory attributes of traditional mayonnaise. Its texture results from the dense packing of fat droplets. Reducing fat decreases droplet density, weakening emulsion stability and increasing water content, which lowers viscosity and firmness. To address this, techniques such as water-in-oil-in-water (W/O/W) emulsions have been developed. These systems trap water droplets inside fat droplets, mimicking the creamy structure of traditional mayonnaise with less fat. In addition to structural design, fat replacers are used to restore the desired properties in low-fat mayonnaise [5,11,12]. Hydrocolloids, for example, increase viscosity and stability by reducing droplet coalescence. Effective fat replacers must be safe, non-laxative, affordable, and able to replicate the mouthfeel of fat. They fall into three categories: fat-based substitutes, and carbohydrate- or protein-based mimetics. While mimetics cannot fully replicate fat, they provide similar textures and contribute beneficial nutrients such as fibers and proteins [5,7,8,9].

Quality issues in mayonnaise are primarily related to storage and deterioration, both of which are influenced by emulsion stability and oil quality. While commercially processed mayonnaise remains stable for a reasonable period (six months or more), it is classified as a semi-perishable product [13]. The term emulsion stability is widely used to describe an emulsion’s ability to resist changes in its properties over time. Instability can arise from physical changes (alterations in the relative positioning of components) or chemical changes (modifications in the chemical composition of the components) [12]. This is the reason why we studied physicochemical and lipid oxidation stability in this work. Egg yolk acts as an emulsifier in mayonnaise O/W emulsions [9]. Its proteins (livetins, lipoproteins, HDLs, LDLs, and phosvitin) are mainly responsible for emulsion stability, especially at low pH. Although yolk also contains phospholipids and cholesterol, proteins dominate its emulsifying properties. Interactions among these components affect emulsification, and some studies suggest that the emulsifying ability of LDLs is largely due to their apoproteins rather than their polar lipids [14,15]. Therefore, stability refers to maintaining a product’s appearance, texture, and flavor throughout its intended shelf life. From an emulsion science perspective, this primarily involves preventing droplet coalescence, flocculation, and/or creaming. However, chemical or biochemical changes, such as oxidation or hydrolysis reactions, can also lead to quality deterioration [16]. Lipid oxidation leads to the development of undesirable flavors (rancidity) and the formation of potentially toxic reaction products. Additionally, it can contribute to the physical instability of certain emulsions. Notably, lipids within emulsions have a large surface area in contact with the aqueous phase, accelerating the formation of reactive oxygen species and increasing the degradation rate of unsaturated fatty acids. This type of deterioration is a major challenge in food preservation and processing [17]. Many lipid oxidation products act as surfactants and can interact with the interfacial layer surrounding the droplets. If they disrupt the interfacial membrane, coalescence may occur, further compromising emulsion stability [12]. One of the most common strategies to inhibit lipid oxidation and preserve emulsion quality is the addition of antioxidants [18]. The most commonly used antioxidants for protecting fats and oils in food products are phenolic antioxidants, such as butylated hydroxyanisole (BHA), butylated hydroxytoluene (BHT), tertiary butylhydroquinone (TBHQ), and propyl gallate (PG). These synthetic antioxidants are often preferred in industrial emulsion formulations due to their higher antioxidant capacity compared to some natural alternatives and their lower cost [18]. Another important factor is the microbiological stability; an acidic environment with a pH range of 3.0 to 4.5, combined with the preservative effect of undissociated acetic acid (typically added in the form of vinegar), lactic acid, or other weak acids, is essential for ensuring it. These acids also contribute to its characteristic acidic taste [9].

In this context, the objective of this study is to formulate a reduced-fat mayonnaise-type sauce with physicochemical properties similar to commercially available reduced-fat versions, while containing a lower oil content (≤30%). This aims to further reduce calorie intake without compromising the typical characteristics of such products, an area in which no previous studies have been published. The strategy involves formulating reduced-fat mayonnaise-type sauces, which will be compared to a commercial sample in terms of physicochemical properties, such as rheological properties, color, phase separation stability, particle size, and oxidative stability. Additionally, the microstructure of the mayonnaise-type sauce will be correlated with its physicochemical properties. Therefore, this study systematically assesses the combined effects of oil type, fat level, and antioxidant presence on the structural and oxidative behavior of reduced-fat mayonnaise-type sauce. The findings provide a foundation for future work on clean-label reformulations using natural antioxidants and further fat reduction strategies.

## 2. Materials and Methods

### 2.1. Materials

For the formulation of the mayonnaise-type sauces, commercial high-oleic sunflower oil (COUSA) and canola oil (PURILEV) without added synthetic antioxidants were used, along with pasteurized liquid egg yolk (commercial, MARUBY S.A.), guar gum (commercial, Waltary S.A.), white alcohol vinegar (commercial, 4% acidity), refined white sugar (commercial), sodium chloride (for ACS analysis, Dorwil, Argentina), sodium benzoate (Anedra, Research ag, Argentina), sodium azide (Fluka, Charlotte, NC, USA), butylated hydroxyanisole (BHA, Darmstadt, Germany), and butylated hydroxytoluene (BHT, Darmstadt, Germany). The pasteurized liquid egg yolk was frozen at −18 °C and then lyophilized for preservation throughout the study.

The commercial reduced-fat mayonnaise used in this study had the following nutritional information per 100 g of product: 7.3 g of carbohydrates, 1.5 g of proteins, and 24.0 g of lipids. Ingredients: water, sunflower oil (BHT as antioxidant), sugar, corn starch, salt, vinegar, powdered egg yolk, powdered whole egg, lemon juice (0.3%), stabilizer: xanthan gum, acidulant: citric acid, glucono delta-lactone, preservative: potassium sorbate, sequestrant: disodium calcium EDTA, antioxidant: BHT, BHA, colorant: beta-carotene, flavoring identical to natural.

### 2.2. Determination of the Centesimal Composition of Egg Yolk

The moisture, protein (determined using the Kjeldahl method, Nx6.25), and ash (obtained through dry incineration at 550 °C) contents of the pasteurized liquid egg yolk were measured following AOAC procedures [19]. Lipid content was analyzed through extraction with a hexane/isopropanol mixture (3:2 *v*/*v*) according to the method of Hara and Radin [20]. Carbohydrate content was determined by difference.

### 2.3. Preparation of Reduced-Fat Mayonnaise-Type Sauces

Three formulations of mayonnaise-type sauces with different oil percentages (10%, 15%, and 30%) were prepared, based on the formulations by Wendin et al. [21]. The content of egg yolk, sodium chloride, sodium benzoate and sucrose was proposed by Wendin et al. The concentration of Guar gum (GG) was varied, because as the oil content decreases, the viscosity decreases due to the reduced number of oil droplets in the system. Therefore, to counteract this phenomenon, the GG concentration must be increased (Table 1). To ensure the preservation of the samples at room temperature throughout the experiment, sodium azide (0.02 g/100 g water) was added as the samples would not be used for sensory evaluation. Additionally, vinegar was incorporated into an amount of 5% to ensure the samples had a pH of 4.0 or lower after preparation.

Before elaborating the mayonnaise-type sauce, the freeze-dried egg yolk was hydrated with distilled water, considering the moisture content of liquid egg yolk and the percentage of yolk in the egg (approximately 20%). The water was added to the lyophilized egg yolk (LEY) by homogenization using an Ultra-Turrax IKA T25 (IKA-Labortechnik, GmbH & Co., Staufen, Germany) with a dispersing element S25N-10G for 5 min at a speed of 9000 rpm.

In a container, the required amount of water according to the corresponding formulation (F1, F2, or F3) from Table 1 was added. The ingredients were then added, and the mixture was continuously stirred at 11,500 rpm for 10 min using a 600 W Philips mixer (Philips & Co., Eindhoven, The Netherlands): sodium azide, sucrose, guar gum, rehydrated freeze-dried egg yolk, vinegar, and sodium chloride.

The samples used to evaluate oxidative stability were prepared with canola or high-oleic oil to evaluate the influence of the fatty acid composition of the oil and with or without the addition of the antioxidant mixture (BHA and BHT), to evaluate their efficiency in preventing oxidation of the oils. The antioxidants were selected based on their solubility and use in emulsions. A mixture of BHA and BHT is used because they act synergistically to provide greater antioxidant activity than when used separately [22]. A concentration of 50 ppm of each antioxidant is used, complying with the lowest maximum limit of both additives (100 ppm BHT) established by Uruguayan Food Regulations [4]. Canola and high-oleic oils were selected due to their common use in Uruguay and distinct fatty acid profiles. Canola oil, higher in polyunsaturated fats, is more prone to oxidation, while high-oleic sunflower oil, richer in monounsaturated fats, offers greater oxidative stability. Their inclusion allowed for assessing the impact of oil type on the stability and structure of reduced-fat mayonnaise-type sauce formulations. BHA and BHT were selected as well-established synthetic antioxidants to provide a standardized baseline for oxidative stability under controlled conditions. Their widespread use and well-characterized properties enable consistent comparison across formulations, though the growing interest in natural, clean-label alternatives is acknowledged. The mixture of oil and antioxidants is placed in an ultrasonic bath (Ney Ultrasonik 57 H) at 400 W for 10 min to solubilize the antioxidants.

The oil phase is then added to the aqueous mixture and homogenized using an Ultra-Turrax with a dispersing element S25N-18G at 12,000 rpm for 5 min. After the emulsification process, the mayonnaise-type sauce is checked to ensure it has a homogeneous appearance and that the oil is fully incorporated.

The nomenclature used for commercial mayonnaise is MH. For the formulations, the name starts with ‘M,’ followed by the type of oil (high-oleic, HO, or canola, C), the oil content (10%, 15%, or 30%), and, if antioxidants are included, an ‘A’ is added at the end. For example, formulation 1 in Table 1 with antioxidants would be labeled MHO-10%–A, and without antioxidant, it would be MHO-10%.

All the formulations were prepared in triplicate and stored at room temperature, protected from the light.

### 2.4. Determination of Flow and Rheological Behavior

The flow curves of the different formulations were compared to those of the reduced-fat commercial mayonnaise (MH) to select the formulations for further study. Flow curves were obtained using a Thermo Haake VT500 viscometer (Thermo Fisher, Anröchte, Germany). The temperature was maintained constant at 20 ± 1 °C using water recirculation from a water bath (HAAKE E8, Thermo Fisher Scientific, Newington, CT, USA). A sensor system with concentric cylinders’ geometry (MV1) was used, with a gap of 0.96 mm between the outer and inner cylinders. From a rheological perspective, the mayonnaises are expected to exhibit thixotropy. To prove this behavior shear stress measurements were taken as a function of shear rate, the shear rate was increased from 0.083 to 5.00 s^−1^ and then decreased from 5.00 to 0.083 s^−1^. Since all sample curves exhibited hysteresis (indicating thixotropy), a standardized measurement procedure was followed. After loading the sample into the viscometer, it was left to rest for 5 min before starting the measurements (allowing the sample to adapt to the measurement conditions, which were the same for all samples), with 3 min between each change in shear rate.

Additionally, mayonnaises generally fit the Herschel–Bulkley model. Therefore, they are viscoelastic fluids that exhibit solid-like behavior until the shear stress exceeds the yield stress (τ_0_). Once this value is surpassed, they exhibit pseudoplastic behavior, flowing more easily as the shear rate increases. The mathematical model of Herschel–Bulkley is given in Equation (1):

(1) $$\tau =\tau_{0}+K*{\dot{\gamma }}^{n}$$

τ is the shear stress and $\dot{\gamma }$ is the shear rate. τ_0_ is a rheological parameter related to the level of internal structure that the material possesses, which must be broken down for it to flow [23]. The parameter K in Equation (1) represents the consistency index, and n represents the flow behavior index, with a value less than 1. If τ_0_ is zero, Equation (1) transforms into the Power Law (Equation (2)), and the flow behavior becomes pseudoplastic [24].

(2) $$\tau =K*{\dot{\gamma }}^{n}$$

The rheological behavior was determined using an Anton Paar Physica MCR 301 rheometer (Anton Paar GmbH, Graz, Austria) with a PP50/TG-SN11173 measuring system (plate-plate geometry) and a gap of 1 mm. The flow curve (shear stress vs. shear rate) was obtained, and K, n, and τ_0_ were determined by applying the Power Law or the Herchel–Bulkley model as appropriate.

A stress sweep was performed at a frequency of 1 Hz, and G’ (elastic modulus) and G’’ (viscous modulus) were determined. The limit of the LVER (linear viscoelastic region), τ_y_, yield point, where G’ remains constant, was calculated by selecting a tolerance of ±5% of the plateau value from the G’ graph, in accordance with ISO 6721-10 and EN 14770 standards [25]. Additionally, the flow point τ_f_ (shear stress value when G’ = G’’) and the loss factor, tanδ = G’’/G’, were determined to assess the “viscoelastic ratio” of the sample’s behavior, where δ represents the phase angle [25].

### 2.5. Phase Separation Stability

The stability of the formulations was evaluated using a vertical optical analyzer (Turbiscan Classic MA2000, Formulaction, Toulouse, France). The emulsions were placed in a cylindrical glass cell (80 mm) to record the Backscattering (BS) profile as a function of height of the cell. Measurements were taken weekly for 3 months. As a stability parameter, the variation in Backscattering, |∆BS|, was considered between the measurement at three months and the initial time (t_0_) for the 20–50 mm region of the cell containing the sample. The results show the variation in the profile over time [26].

### 2.6. Microstructure Determination

The microstructure was analyzed using an OLYMPUS CX23 optical microscope (Olympus, Tokyo, Japan) with a 40× magnification [27]. The samples were examined at the time of preparation and after 3 months of storage.

### 2.7. Particle Size Measurement

In this case, the particle size distribution was determined by laser light diffraction and polarized light scattering using a Beckman-Coulter LS-230 particle size analyzer (Coulter Electronics Ltd., Luton, UK). The parameters D(3,2) (Sauter diameter) and D(4,3) (De Brouckere diameter) were obtained [27].

### 2.8. pH Measurement

The pH of the formulations after preparation was determined directly with an insertion-type pH electrode at 25 °C, using an OAKTON/pH 700 pH-meter calibrated with 7.00 ± 0.02 and 4.00 ± 0.02 buffers (Hydrion, Micro Essencial laboratory, New York, NY, USA).

### 2.9. Color Measurement

Color measurements were made in the cylindrical glass cells where the samples were stored to measure stability (2.6) weekly for 3 months using a Konica Minolta CM-2300d colorimeter (Konica Minolta, Inc., Tokyo, Japan). The parameters L* (luminosity), a* (red-green coordinate), and b* (yellow-blue coordinate) were obtained.

### 2.10. Determination of Fatty Acid Composition and Tocopherols Content of High-Oleic Sunflower and Canola Oils

The fatty acid composition was determined by first derivatizing the oils according to the IUPAC 2.301 method [28]. The resulting methyl esters were analyzed using gas chromatography (GC). Fatty acid lipid profile analysis was conducted following AOCS Ce 1c-89 and AOCS Ce 1f-96 methods [29], employing a Shimadzu 2014 system equipped with an FID detector and a Supelco SP-2560 capillary column. Peak identification was performed using a Supelco standard mixture of fatty acid methyl esters (C4–C24) containing all the methyl esters of the fatty acids relevant to the analysis.

Subsequently, its inherent stability, as outlined by Gunstone and Hilditch [30], was assessed to indicate the oil’s susceptibility to oxidation. In addition, oxidability was evaluated following the method of Cosgrove et al. [31], which also enables the estimation of the “vulnerability” of a fatty acid mixture to oxidation.

The fatty acid composition of high-oleic sunflower and canola oils and their inherent stability and oxidability are shown in Table 2.

The determination of the tocopherol content was conducted by high-performance liquid chromatography (HPLC) using a Shimadzu Model 20 system with a fluorescence detector (RF Model 20A XS) and a Macherey-Nagel C18 column (250 × 4.6 mm, 100 μm) at 40 °C. Briefly, 30 mg of oil was diluted in 1 mL of HPLC-grade isopropanol, and a 50 μL aliquot was injected. Chromatograms were recorded (λex = 290 nm, λem = 330 nm) using an initial mobile phase of acetonitrile (18%), methanol (13%), water with 0.5% acetic acid (69%), and isopropanol (0%), following Andrikopoulos et al. [32]. Tocopherols (α, β, γ, δ) were quantified against calibration curves prepared with Sigma Aldrich standards.

### 2.11. Study of Oxidative Stability

This study was conducted under accelerated storage conditions by maintaining the samples of formulations with and without antioxidants in a forced convection oven at 65 °C for 3 days. The study utilized accelerated storage conditions to simulate long-term storage at room temperature, with each day at 65 °C approximating one month of ambient storage [30]. This 3-day period thus represents about three months of real-time storage, enabling a rapid assessment of oxidative stability and shelf-life-related changes without the need for prolonged testing [33]. Subsequently, oil was extracted from the samples (method described in 2.2) at time 0 and after 3 days, and its degree of oxidation was assessed by determining the induction period (IP) using the Rancimat method with the 873 Biodiesel Rancimat (Metrohm, Switzerland) at 100 °C and an airflow of 20 L/h.

### 2.12. Statistical Analysis

All analyses were performed at least in triplicate. The data are expressed as the mean ± 2 x standard deviation. Analysis of variance and Tukey’s comparison test were performed using Infostat software, version 2020e. For the determination of parameters k and n, Statistica 8.0 software was used. A significance level of 0.05 was applied.

## 3. Results and Discussion

### 3.1. Proximal Composition of Pasteurized Liquid Egg Yolk

Table 3 presents the results for moisture, lipids, proteins, and ash content in pasteurized liquid egg yolk. The obtained results are consistent with those reported by Koppmann [34], Ecuatorian Food Composition Table [35] and the Uruguayan Food Composition Table [36].

### 3.2. Formulation, Flow Behavior, and Rheological Characterization of Mayonnaise-Type Sauce

Texture is the sensory perception of a product’s structure. Its rheological properties play a crucial role in creating a creamy mouthfeel, although other factors may also contribute. Among other factors, oil content gives mayonnaise a semi-solid behavior that affects its rheological properties, which in turn influence the product’s perceived texture and flavor [37,38]. The properties of the aqueous phase can also affect the texture and mouthfeel of food emulsions. The presence of biopolymers, particularly those with thickening or gelling properties, plays a key role. The viscosity of emulsions is directly proportional to that of the continuous phase; therefore, any component in the aqueous phase that enhances its viscosity will impact the overall rheology and textural properties of the system [11].

#### 3.2.1. Formulation of Mayonnaise-Type Sauces

Different mayonnaise-type sauce formulations were tested to achieve flow properties similar to those of commercial mayonnaise. GG was used to control the viscosity of the continuous phase, adjusting its concentration within a range of 0.75–1.55%. Since the oil content varied across formulations, the proportion of oil droplets also changed, leading to variations in rheological behavior.

**Figure 1:**
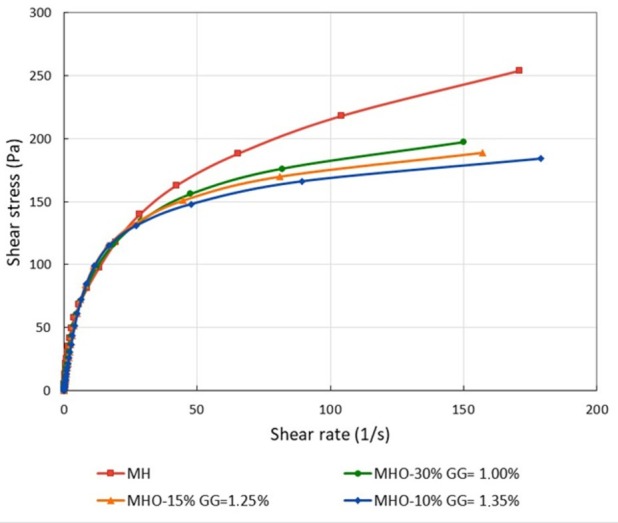
Shear stress (Pa) vs. shear rate (s^−1^) for MH, MHO-10%, MHO-15%, and MHO-30% at the selected GG concentrations (1.35%, 1.25%, and 1.00%, respectively).

Shear stress (τ) vs. shear rate (${\dot{\gamma }}^{n}$) curves were compared for different formulations (MHO-10%, MHO-15%, MHO-30%) and MH. All analyzed samples exhibited thixotropic behavior, so two curves were recorded: in the first, the shear rate was increased (upward curve) to values between 151 and 179 s^−1^ (depending on the sample and GG concentration), and in the second, it was decreased back to 0 (downward curve). The results showed that the flow curves of the MHO-10%, MHO-15%, and MHO-30% formulations closely resembled that of commercial mayonnaise at GG concentrations of 1.35%, 1.25%, and 1.00%, respectively (Figure 1).

#### 3.2.2. Flow Behavior of Mayonnaise-Type Sauce

Experimental vs. theoretical shear stress (τ) values were plotted. The experimental τ values were derived from the shear stress vs. shear rate curve, while the theoretical τ values were calculated using the corresponding equation for the Power Law or Herschel–Bulkley models, which allowed the determination of K, n, and τ_0_. The coefficient of determination (r^2^) was 0.999 for the Power Law and 0.996 for the Herschel–Bulkley model, indicating a better fit with the Power Law.

Rodrigues do Carmo et al. [39] observed that for a mayonnaise with 40% oil content, both the Bingham and Power Law models provided good fits under most conditions. However, the Herschel–Bulkley model was the only one that predicted the flow curves with the best fit (r^2^ = 0.999), which is expected for this type of product, as it should exhibit a yield stress threshold and pseudoplastic behavior, as noted by García Félix [40].

Changes in thickness in food are generally associated by consumers with changes in the product’s viscous behavior. As a result, predictive correlations have been developed between consistency and rheological properties of foods, approximating the mouth geometry to a well-known one, typically resembling two parallel plates [41].

Additionally, Su et al. [42] studied the fit of reduced-oil mayonnaise to the Herschel–Bulkley model using various gums as thickeners (guar gum and xanthan gum). They observed that as the amount of guar gum increased, the flow behavior index (n) decreased. This same trend was observed in this study, with n for MHO-10% < MHO-15% < MHO-30%, which suggests that as oil content decreased and guar gum content increased, the flow behavior deviated more from Newtonian. As shown in Table 4, all the samples presented significant differences (*p* > 0.05). For all the mayonnaise-type sauces, an n value < 1 was obtained, which means they exhibit pseudoplastic behavior, flowing more easily as the deformation rate increases. The highest n value was observed for the commercial mayonnaise, indicating that greater effort is required to make it flow at the same deformation rate.

Regarding the K index, significant differences were found between the commercial mayonnaise and the MHO-10% formulation. In contrast, the MHO-15% and MHO-30% formulations showed no significant differences either between them or with the other samples. This is because the formulations were designed so that the addition of GG would compensate for the reduction in oil content. If viscosity had not been adjusted with GG, an increase in the oil fraction would have likely resulted in a significant rise in the viscosity of the emulsions, making them more resistant to flow [43].

Ma and Boye [9] stated that oil content is a key factor in determining the rheological behavior of emulsions. An increase in oil content leads to higher K and n values. This is because lipoproteins adsorbed onto the oil droplets interact with each other, forming a more compact structure that requires more effort to flow.

According to previous studies by Liu et al. [16], the large surface area of contact between oil droplets leads to significant frictional forces that resist the flow of the emulsion when subjected to stress, resulting in an increase in its viscosity. As the particle diameter decreases, the contact area between droplets increases, thereby raising the viscosity. In this case, we observe that K shows no significant differences between MH, MHO-15%, and MHO-30% (Table 4), and this same trend is seen for D(4,3) (Table 6). This also explains the flow behavior of the formulations and the commercial mayonnaise.

#### 3.2.3. Rheological Characterization of Mayonnaise-Type Sauce

As shown in Table 5, for all cases, G’ is greater than G’’ (G’ > G’’) within the linear viscoelastic region (LVER), indicating that all the formulations and the mayonnaise analyzed exhibit a gel-like structure and can be considered viscoelastic solid materials [25,43].

Additionally, it can be concluded that as the oil content increases, so does the value of G’. This suggests the formation of a structure connected by flocculated oil droplets [43]. Mayonnaise-like fluids exhibit linear viscoelastic behavior, which may reflect the nature of the intermolecular forces between the lipoproteins adsorbed around the oil droplets [44].

Egg yolk is composed of phospholipids (lecithin), proteins, and lipoproteins (lipovitellin and livetin). These components give mayonnaise the ability to flocculate, enhancing the emulsion’s texture. Lipovitellin and livetin are believed to be the molecules most responsible for promoting the emulsion-forming properties. The pH of the emulsion plays a crucial role in its stability. Viscoelasticity and stability are expected to be higher when the pH is near the average isoelectric point of the egg yolk proteins [41]. Bautista Villarreal et al. [14] determined through zeta potential measurements that the average isoelectric point of egg yolk is 4.6. They found that viscoelasticity was highest at a pH of 3.9 [41].

In the formulations, the storage modulus is always higher than the loss modulus within the LVER, although the differences are smaller for the emulsions with 10% and 15% oil. This is because increasing the oil content enhances the elastic properties of the emulsion [45].

All formulations showed higher storage modulus (G’) values and lower loss modulus (G’’) values, indicating a gel-like behavior for the emulsions (Table 6). The tan δ values (G’’/G’) were below 0.5 at 1 Hz, confirming that the emulsions behaved as weak physical gels, predominantly elastic. This behavior was also observed by Zhang et al., and Katsaros et al. [2,43].

**Table 6:** Particle Size Measurements at Initial Time and After 3 Months, and |∆BS| for the Different Formulations and Commercial Mayonnaise.

Sample	D(3,2) (µm) Initial	D(3,2) (µm) 3 Months	D(4,3) (µm) Initial	D(4,3) (µm) 3 Months	|∆BS| (%)
MH	(4.25 ± 0.29) ^b,A^	(4.06 ± 0.31) ^c,A^	(15.01 ± 0.66) ^b,A^	(15.50 ± 0.83) ^b,A^	(0.54 ± 0.17) ^b^
MHO-10%	(4.03 ± 0.08) ^b,A^	(3.67 ± 0.32) ^b,A^	(23.55 ± 1.37) ^c,A^	(21.91 ± 0.93) ^d,A^	(4.02 ± 0.37) ^a^
MHO-15%	(3.24 ± 0.12) ^a,A^	(3.19 ± 0.23) ^a,A^	(13.97 ± 5.26) ^a,b,A^	(17.66 ± 0.75) ^c,A^	(5.16 ± 0.19) ^a^
MHO-30%	(3.29 ± 0.48) ^a,A^	(2.84 ± 0.40) ^a,A^	(11.15 ± 2.70) ^a,A^	(10.24 ± 2.79) ^a,A^	(5.09 ± 0.83) ^a^

Values in the same column marked with the same lowercase letter are not significantly different (*p* > 0.05). Similarly, for each parameter, values in the same row marked with the same uppercase letter are not significantly different (*p* > 0.05).

**Figure 2:**
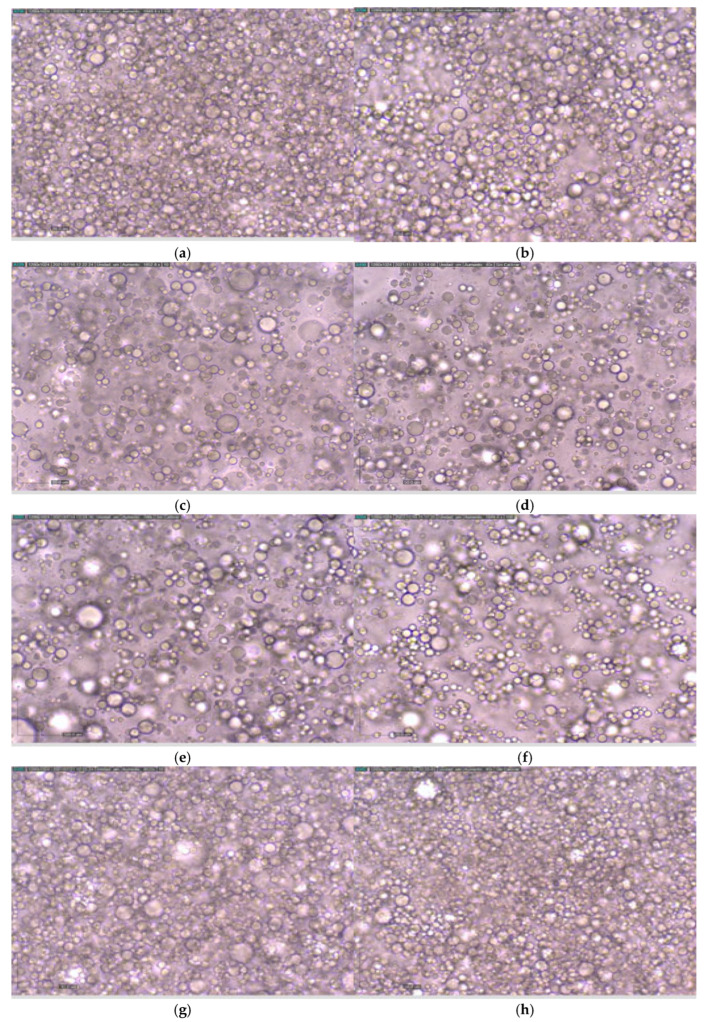
Images obtained under a microscope with 40× magnification for the different formulations and the commercial mayonnaise at the initial time and after 3 months of storage. At initial time: (**a**) MH, (**c**) MHO-10%, (**e**) MHO-15%, (**g**) MHO-30%. After 3 months of storage: (**b**) MH, (**d**) MHO-10%, (**f**) MHO-15%, (**h**) MHO-30%.

Significant differences were found between commercial mayonnaise (MH) and the other formulations in the loss factor. The commercial mayonnaise exhibited the lowest loss factor, indicating a greater elastic behavior. This can be explained by the observations in the micrographs (Figure 2), where the compact packing of oil droplets in a network structure is responsible for the elastic properties and resistance to deformation of the emulsion [16]. Liu et al. and Patil and Benjakul [16,37] observed that mayonnaises with a more compact structure showed higher G’ values because of increased hydrophobic interactions between the oil droplets, requiring more effort to make the mayonnaise flow. A similar trend is observed here: commercial mayonnaise has the highest G’ and the most compact structure (Figure 2 and Table 5).

Table 5 shows that the samples with lower oil content have higher flow points. This can be explained by the higher guar gum content in the formulations; both MHO-10% and MHO-15% contain 1.25% and 1.35%, respectively. A flow point at higher shear indicates that the viscous component will dominate, and the sample will flow [25].

According to Ma and Barbosa-Cánovas [46], thickeners in solution at concentrations of 0.5% or lower form aggregates through hydrogen bonding, strengthening the structure. At higher concentrations, they form a viscoelastic structure to stabilize the emulsion through hydrogen bonds [47]. This structure increases the consistency of the mayonnaise, thus raising its flow point. This could explain the higher flow points for MHO-10% and MHO-15%.

### 3.3. Microstructure, Particle Size, and Stability

#### 3.3.1. Microstructure

Figure 2 presents micrographs of the different formulations at the initial time and after 3 months of storage. No differences in droplet size were observed at different times for MH, MHO-15%, MHO-30%, and MHO-10%, as will be discussed later with the D(4,3) and D(3,2) parameters.

MHO-30% shows the highest droplet density and clustering compared to the other formulations due to its higher oil content. Commercial mayonnaise shows more homogeneous droplet sizes compared to the formulations, which exhibit a multimodal distribution. This can be explained by the fact that it is often produced using a combination of a high-shear mixer to form a coarse emulsion and a homogenizer (colloidal mill or high-pressure valve homogenizer) to reduce droplet size and achieve ingredient homogenization. This equipment ensures that the oil droplets are reduced in size and distributed uniformly. The colloidal mill allows for adjustment of the gap between the rotor and stator, forcing the sample through this space, which results in the formation of small oil droplets [12,48]. Previous studies by Worrasinchai et al. [49] state that mayonnaises with lower oil content exhibit larger spaces surrounded by aggregates of oil droplets, a feature that aligns with what is observed for MHO-10% and MHO-15%, where flocs can be seen (Figure 2).

#### 3.3.2. Particle Size

The droplet size is another important parameter because of its ability to influence the appearance, texture, and flavor profile of the product [37]. Table 6 presents the values for D(3,2) and D(4,3) at the initial time and after 3 months of storage. The D(4,3) values were significantly higher than the D(3,2) values, indicating that the emulsions were polydisperse. This phenomenon becomes more pronounced as the D(4,3) value increases in relation to D(3,2) [50,51].

At time zero, D(3,2) does not show significant differences between the commercial mayonnaise and the MHO-10% formulation, nor between the MHO-15% and MHO-30% formulations. On the other hand, for D(4,3), the MHO-15% formulation does not show significant differences when compared to the commercial mayonnaise and the MHO-30% formulation.

Table 6 also shows that formulations with a higher oil content tend to have smaller particle diameters, as the oil droplets have less space to pack together.

Patil and Benjakul [37] studied the particle size of different mayonnaises at time zero and after three months, observing that both D(3,2) and D(4,3) increased over time. This was attributed to destabilization processes such as coalescence and flocculation, which lead to an increase in particle size. However, in this study, no significant changes were observed over time for the MH, MHO-15%, and MHO-30%-SA samples, which aligns with the micrograph observations.

#### 3.3.3. Storage Stability

Stability involves preventing droplet coalescence, flocculation, and/or the formation of a cream phase. Droplet flocculation in dressings depends on the product. While droplet flocculation is generally considered undesirable for stability, it can be beneficial in certain dressings. In mayonnaise, the driving force is due to electrostatic attraction between droplets. Flocculation increases the viscosity of the emulsion and can even give it gel-like properties due to the formation of a three-dimensional network of aggregated particles, as seen in the micrographs in Figure 2.

Preventing coalescence is crucial in many dressings, as it leads to the formation of free oil on the product’s surface, which is generally undesirable. The most important stabilizing force in a given dressing depends on the type of emulsifier used (e.g., protein, polysaccharide, surfactant, or colloidal particles) and the environmental conditions (e.g., pH and ionic strength). Most dressings contain relatively high salt concentrations, which tightly regulate electrostatic repulsive forces. As a result, steric repulsion is expected to be the dominant stabilization mechanism in many systems, particularly those stabilized by relatively thick layers of biopolymers or particles. Particle stabilization plays a key role in systems where colloidal particles are adsorbed onto interfacial layers, such as egg yolk, spices, or starch granules [12].

Creaming is generally not a concern in dressings with high fat content (>50–60%) because the droplets are so densely packed that they cannot move—this is the case for mayonnaise and spoonable salad dressings. In lower-fat products, creaming is typically prevented by adding thickening or gelling agents to the aqueous phase to slow droplet movement, such as gums or starches. In this study, GG was added for this purpose [12].

The stability of egg yolk emulsions is primarily due to the presence of granules (which contain HDL, phosvitin, and LDL). This fraction has a higher protein content than the plasma, which may contribute to the formation of a thick protein layer at the droplet interface. This, in turn, ensures emulsion stability by increasing both electrostatic and steric repulsion and allowing cross-linking between droplets [52].

The stability of the formulations and commercial mayonnaise was assessed by monitoring changes in backscattering over time, placing the samples in a cell (see Section 2.5). According to Huck-Iriart et al. [53], creaming leads to a variation in concentration between the top and bottom of the cell. Droplets move upward due to their lower density compared to the surrounding liquid. When profiles are displayed in reference mode, a BS peak appears in the lower region (0–20 mm) if destabilization by creaming is occurring. In the case of flocculation, the analysis focuses on the middle section of the cell (20–50 mm), which is considered unaffected by creaming.

**Figure 3:**
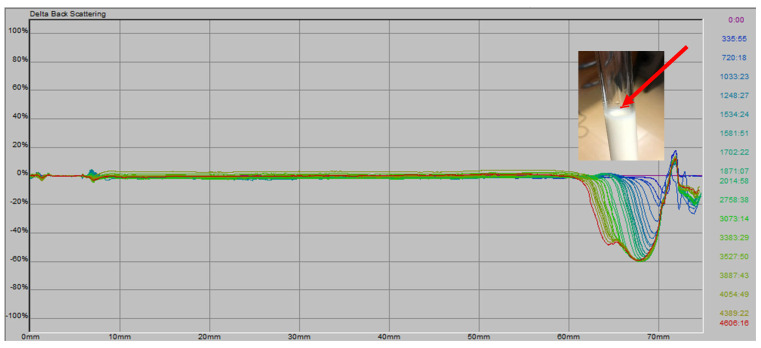
∆BS (%) vs. cell length (mm) and observed volume reduction for the different formulations. The red arrow indicates the initial sample level at time 0.

In Figure 3, the behavior of BS as a function of time and tube height is shown for all samples. Minimal variation is observed over time up to approximately 60 mm, where a decrease in BS values occurs. This can be explained by the sample preparation method (using a mixer and Ultra-Turrax) and the filling of the cells, during which air is incorporated. Over time, these air bubbles dissipate, causing a contraction in sample volume [40]. This phenomenon is visible as a volume reduction, as shown in Figure 3. The red arrow indicates the initial sample level at time 0.

Commercial mayonnaise has a minimum shelf life of six months, during which no destabilization is observed. The reference value of |∆BS| = 0.54% from MH is used to compare with the values obtained for the different formulations.

For MHO-10%, MHO-15%, and MHO-30%, the |∆BS| variation is significantly higher than that of commercial mayonnaise (Table 5).

Biller et al. [54] attribute changes in BS to variations in mayonnaise particle size during storage. However, in this study (Table 6), no significant differences were observed in particle size (D(3,2) and D(4,3)) between t = 0 days and t = 3 months for any of the samples. Therefore, this parameter does not affect sample stability within the measurement period, which aligns with the low |∆BS| values.

MHO-10% has a higher D(4,3) than MH, while MHO-30% has a lower value, with no significant differences between MHO-15% and MH (Table 6). Therefore, the difference in |∆BS| values between the formulations and MH cannot be attributed to droplet size differences. Instead, it is likely due to variations in the composition of the continuous phase.

MH contains corn starch and xanthan gum as thickening agents. The physicochemical mechanism responsible for the rheological functionality of each hydrocolloid is determined by the molecular structure of its polymer [55]. The primary structure of xanthan gum consists of a β-(1–4)-D-glucose backbone substituted with trisaccharide side chains at the C-3 positions of alternating glucose residues, which carry negative charges. In aqueous solutions at relatively low temperatures, xanthan gum is believed to exist as rigid, extended molecules with a predominantly helical structure. Under suitable solution conditions, the helical regions of different molecules can associate, leading to the formation of a weak gel [12]. This gel traps and immobilizes oil droplets, creating an effective yield stress that is sufficient to counteract the buoyant forces acting on individual droplets [41].

This behavior has not been reported for GG, suggesting that the presence of xanthan gum in commercial mayonnaise may explain its greater stability. GG consists of a β(1,4)-linked mannose backbone with α(1,6)-linked galactose branches, with a mannose-to-galactose ratio of 2:1. Due to its high degree of substitution and lack of charge, it is unlikely to form a gel.

### 3.4. pH

Commercially elaborated dressings and mayonnaise are generally considered safe because foodborne pathogens do not grow under the acidic conditions present in these products (i.e., pH < 4.4). Additionally, the organic acids used in these formulations, such as acetic acid, are particularly effective in preventing microbial growth or eliminating introduced bacteria. Many dressings also contain preservatives and antimicrobial agents, such as sodium benzoate or benzoic acid, to further inhibit bacterial growth [12].

Table 7 presents the initial pH values of the mayonnaise-type sauce samples. All samples have a pH of 4 or lower, meeting the physicochemical requirement established by the RBN [4] and remaining below the safety threshold of pH < 4.4 mentioned earlier. To ensure product safety, pH should also be measured at the end of its shelf life, and the microbiological requirements set by the RBN [4] must be met.

### 3.5. Color

Table 7 presents the results of the L*a*b* parameters for the different mayonnaise-type sauce samples, with no significant differences observed between the measurements taken at the initial time and after three months for the same sample.

For the L* parameter, no significant differences were observed between the different formulations and the commercial mayonnaise.

L* represents the opacity of the emulsion and is determined by the concentration, size, and refractive index of the oil droplets. For O/W emulsions, brightness increases sharply as the oil content rises (from 0% to 5% by weight) but then increases more gradually as the oil percentage also increases due to greater light scattering by the oil droplets, reaching an asymptote at approximately 10% [56]. This phenomenon may have implications for the creation of reduced-fat foods, as a decrease in brightness may be associated with an undesirable loss of creaminess [11]. In this case, all the samples analyzed contain oil content equal to or greater than 10%, so the L* values remain constant with no significant differences.

Further, brightness is also affected by the distribution of particle size, as it determines the efficiency of light scattering. This aspect may vary depending on the mayonnaise preparation method and the addition of non-lipid ingredients [56]. In this study, MHO-10% has the highest D(4,3), while MH has a lower value and MHO-30% has the smallest, with no significant differences between MHO-15% and MH. However, no differences were observed in L*, likely because the predominant factor affecting opacity in the samples is the addition of gums rather than the particle size (Table 7).

When it comes to parameters a* and b*, however, significant differences were found between the formulations and the commercial mayonnaise (Table 7).

Regarding the a* parameter, its value remains very close to 0 and shows no significant variation over the 3-month period, indicating minimal impact on the overall color. Yüceer et al. [57] also reported a* values near 0.

Additionally, positive values for the b* parameter indicate the presence of yellow color. For commercial mayonnaise, this value is higher than for the formulations due to the use of β-carotenes as colorants (see Section 2.1) [58].

According to Carcelli et al. [8], a significant reduction in b* (yellow) for reduced-fat mayonnaises can be associated with the increase in brightness and the decrease in oil content, which leads to a color alteration. In the formulations, b* values for MHO-30% are higher than for MHO-10% and MHO-15%. This is due to the addition of thickeners (GG in this case) replacing oil, which is the component contributing to the yellow color.

### 3.6. Fatty Acid Composition of Oils and Oxidative Stability

The fatty acid composition (in %) of sunflower high-oleic and canola oils, as well as the calculated oxidative stability, are shown in Table 2.

Canola oil contains a higher proportion of polyunsaturated fatty acids compared to high-oleic sunflower oil, which corresponds to its higher oxidability and inherent stability, making it more susceptible to oxidation when considering only its fatty acid composition Table 2. However, it should be noted that minor components, such as residual tocopherols (that remain after the refining process) present in commercial oils, can have an antioxidant function [59]. In the study, high-oleic oil showed a higher α-tocopherol content (809.0 ± 0.7 mg/kg) compared to canola oil (208.1 ± 0.2 mg/kg). In contrast, canola oil exhibits much higher levels of β + γ-tocopherols (1211.3 ± 0.9 mg/kg) and δ-tocopherol (57.7 ± 0.2 mg/kg) compared to high-oleic oil (76.2 ± 0.1 mg/kg and 7.0 ± 0.1 mg/kg, respectively). These tocopherol isoforms also contribute to antioxidant activity but differ in their potency and stability. The elevated β + γ and δ tocopherols in canola oil may provide different antioxidant mechanisms, potentially affecting the oil’s oxidative stability and shelf life. This information is essential because of potential synergistic effects, such as the interaction between phosphatidylinositol (found in egg yolk) and tocopherols in reducing lipid oxidation, as well as the interplay between tocopherols and carotenoids [60].

During the storage of mayonnaise, lipid oxidation can occur, which reduces its quality. This process leads to a loss in nutritional value, the development of undesirable off-flavors, and the formation of potentially toxic reaction products. Its progression can be slowed using a variety of strategies, one of which is the incorporation of antioxidants [12].

Table 8 shows the Rancimat induction period (IP) obtained for t = 0 days and t = 3 days of storage at 65 °C. The induction time is a measure of the lipid material’s resistance to oxidation; the shorter the induction time, the more vulnerable the sample is to oxidation [61]. At time 0, the samples MHO-10%, 15%, and 30% and MC-10%, 15%, and 30% without added antioxidants do not show significant differences in IP. However, with the addition of antioxidants, the induction time quadruples for all three formulations, MHO-10%-A, MHO-15%-A, and MHO-30%-A. The addition of antioxidants (BHT + BHA mix) also allowed the mayonnaises stored at 65 °C to have high IP values at t = 0 (7.62, 7.88, and 8.03 for MHO-10%, 15%, and 30%, respectively), providing a protective effect under the accelerated oxidation conditions studied.

The IP values obtained in this study are higher than those reported in the work of Ahmadi-Dastgerdi et al. [62], where TBHQ was incorporated at a concentration of 0.12 mg/mL in mayonnaise, yielding an IP of 4.83 h (Rancimat at 110 °C). The induction times obtained at t = 0 days for the canola and high-oleic oils (Table 8) were similar to those found by Merrill et al. [63], 8.4 h and 16.5 h, and Jimenez et al. [64], 9.5 h and 16.9 h, respectively.

Conversely, the induction times for both oils are higher than the t = 0 values when incorporated into the formulations. This can be attributed to the oxidation occurring during the preparation of the mayonnaise-type sauce, caused by the incorporation of oxygen and the rise in temperature, as part of the mechanical energy from stirring is converted into heat through viscous dissipation [12].

High-oleic oil has a higher induction time than canola oil (Table 8), which is expected due to its fatty acid composition. However, no significant differences were found in the induction times of mayonnaise-type sauce prepared with high-oleic and canola oils for the same oil concentration, which may also be explained by the oxidation occurring during homogenization.

Regarding the induction times obtained for the samples subjected to accelerated rancidity at 65 °C for three days, formulations prepared with canola and sunflower high-oleic oil show significant differences (*p* < 0.05) in their induction times. Canola oil has a higher proportion of polyunsaturated fatty acids, inherent stability, and oxidability compared to high-oleic oil, which results in formulations made with the former undergoing greater deterioration during the treatment due to its higher susceptibility to oxidation.

Overall, these results emphasize that oxidative stability in emulsified systems is multifactorial, influenced not only by oil composition but also by processing, storage, and additive strategies. The inclusion of antioxidants significantly enhances oxidative resistance, but further optimization, such as the use of natural antioxidants or encapsulation techniques, could improve stability in future formulations. In our study, the IPs of the formulated mayonnaise-type sauce were lower than those of the bulk oils, reinforcing the notion that emulsion processing introduces pro-oxidant factors that compromise stability.

## 4. Conclusions

This study successfully developed reduced-fat mayonnaise-type sauce formulations using GG and varying oil levels (10%, 15%, and 30%). The 15% oil formulation (MHO-15%) exhibited rheological and particle size characteristics closest to commercial reduced-fat mayonnaise (MH), with a comparable mean particle size and pseudoplastic flow behavior well described by the power law model.

Despite some differences in BS profiles, all formulations remained physically stable over 3 months, with │ΔBS│ values indicating no signs of destabilization. Rheological analysis confirmed gel-like structures in all samples (G’ > G’’), although MHO-15% showed a lower elastic modulus, suggesting a slightly weaker gel network.

In terms of oxidative stability, formulations with high-oleic sunflower oil had significantly higher induction times compared to those made with canola oil, indicating improved resistance to oxidation. However, sunflower high-oleic oil with added antioxidants should be used, as the induction time increased over time.

Overall, reducing oil content from 30% to 15% maintained desirable texture, structure, and stability, making MHO-15% a promising reduced-fat alternative. Future work should include sensory analysis and microbiological evaluation to validate consumer acceptance and safety.

## Figures and Tables

**Table 1:** Composition of the Different Formulations of Mayonnaise-Type Sauce.

Ingredients (%*w*/*w*)	Wendin et al. [21]		F 1	F 2	F 3
Oil	15.0	30.0	10.0	15.0	30.0
Egg yolk	1.5	3.0	1.5	1.5	3.0
Distilled water	72.3	55.8	76.6	71.7	55.5
Vinegar (%)	4.0	4.0	5.0	5.0	5.0
Sodium chloride	1.4	1.4	1.4	1.4	1.4
Sodium benzoate	0.1	0.1	0.1	0.1	0.1
Guar gum	1.6	1.6	1.4	1.3	1.0
Sucrose	4.0	4.0	4.0	4.0	4.0
Sodium azide	---	---	0.02	0.02	0.02
Potassium benzoate	0.1	0.1	---	---	---

**Table 2:** Fatty Acid Composition of High-Oleic Sunflower and Canola Oils and Their Oxidability and Inherent Stability.

	High-Oleic Sunflower Oil (%)	Canola Oil (%)
16:0	3.5 ± 0.1	4.7 ± 0.3
16:1 n-7	0.1 ± 0.02	0.1 ± 0.01
18:0	2.5 ± 0.1	2.0 ± 0.1
18:1 cis n-9	87.4 ± 1.4	59.4 ± 0.8
18:2 cis n-6	4.7 ± 0.1	22.5 ± 0.5
18:3 cis n-3	0.2 ± 0.03	8.9 ± 0.4
20:0	0.2 ± 0.02	0.1 ± 0.01
20:1 cis n-9	0.3 ± 0.02	0.9 ± 0.02
22:0	0.8 ± 0.02	0.1 ± 0.01
Saturated Fatty Acids	7.3 ± 0.3	6.8 ± 0.2
Monounsaturated Fatty Acids	87.8 ± 1.5	59.5 ± 1.2
Polyunsaturated Fatty Acids	4.9 ± 0.2	31.4 ± 0.7
Oxidability	0.07	0.42
Inherent Stability	1.4	5.1
α tocopherol (mg/kg)	809.0 ± 0.7	208.1 ± 0.2
β + γ tocopherols (mg/kg)	76.2 ± 0.1	1211.3 ± 0.9
δ tocopherol (mg/kg)	7.0 ± 0.1	57.7 ± 0.2

**Table 3:** Proximal Composition of Liquid Egg Yolk Expressed as a Percentage (g/g of Sample) on a Wet Basis.

	Liquid Egg Yolk (% *w*/*w*)	Koppmann [34] (%)	Ecuatorian Food Composition Table [35]	Uruguayan Food Composition Table [36] (%)
Moisture	57.25 ± 0.33	48.0	53.3	52.1
Lipids	28.96 ± 1.40	32.5	26.4	28.7
Proteins	14.51 ± 1.38	17.5	15.9	16.6
Ashes	1.37 ± 0.13	2.0	0.7	2.6

**Table 4:** Results of K and n Obtained by Applying the Power Law and Kokini’s Viscosity.

Sample	K (Pa.s^n^)	n	Kokini’s Viscosity (Pa·s)
MH	33.71 ± 1.35 ^a^	0.382 ± 0.001 ^a^	12.50 ± 0.71 ^a^
MHO-10%	31.35± 0.23 ^b^	0.317 ± 0.001 ^d^	12.15 ± 0.14 ^a^
MHO-15%	32.36 ± 0.52 ^a,b^	0.321 ± 0.003 ^c^	12.55 ± 0.21 ^a^
MHO-30%	32.94 ± 1.01 ^a,b^	0.331 ± 0.002 ^b^	12.62 ± 0.35 ^a^

Values with the same letter in the same column are not significantly different (*p* > 0.05).

**Table 5:** G’ and G’’ in the LVER, Limit of the LVER (τy, Yield Point), Flow Point (τf, Flow Point), and Loss Factor (tan δ) Obtained for the Different Samples.

Sample	G’ (Pa)	G’’ (Pa)	τ_y_ (Pa)	τ_f_ (Pa)	tan δ
MH	194.6 ± 7.7 ^c^	36.7 ± 0.2 ^a^	8.6 ± 0.8 ^a^	76.7 ± 2.2 ^a^	0.189 ± 0.009 ^a^
MHO-10%	82.2 ± 0.7 ^a^	40.6 ± 0.2 ^b^	27.3 ± 0.8 ^c^	106.9 ± 0.7 ^c^	0.494 ± 0.002 ^d^
MHO-15%	88.14 ± 0.01 ^a,b^	41.91 ± 0.14 ^c^	19.6 ± 2.2 ^b^	104.8 ± 0.9 ^c^	0.475 ± 0.002 ^c^
MHO-30%	103.5 ± 1.1 ^b^	44.3 ± 0.3 ^d^	17.5 ± 1.4 ^b^	89.2 ± 1.8 ^b^	0.428 ± 0.002 ^b^

Values with the same letter in the same column are not significantly different (*p* > 0.05).

**Table 7:** Results of L*a*b* and pH for the Different Formulations and Commercial Mayonnaise.

Mayonnaise	Time (Months)	L*	a*	b*	pH
MH	0	(79.61 ± 0.21) ^a^	(1.14 ± 0.01) ^a^	(12.51 ± 0.02) ^a^	3.30 ± 0.01
	3	(76.00 ± 4.60) ^a^	(1.02 ± 0.01) ^a^	(11.25 ± 0.68) ^a^	
MHO-10%	0	(78.73 ± 2.74) ^a^	(−0.01 ± 0.01) ^c^	(4.56 ± 0.07) ^c^	3.92 ± 0.01
	3	(79.70 ± 0.57) ^a^	(−0.09± 0.03) ^c^	(4.53 ± 0.03) ^c^	
MHO-15%	0	(81.15 ± 2.62) ^a^	(−0.01 ± 0.01) ^c^	(4.71 ± 0.25) ^b,c^	3.99 ± 0.01
	3	(80.65 ± 0.13) ^a^	(−0.08 ± 0.01) ^c^	(4.73 ± 0.06) ^b,c^	
MHO-30%	0	(80.87 ± 0.21) ^a^	(0.45 ± 0.03) ^b^	(6.27 ± 0.33) ^b^	4.05 ± 0.01
	3	(82.09 ± 0.71) ^a^	(0.44 ± 0.04) ^b^	(6.56 ± 0.01) ^b^	

Values with the same letter in the same column are not significantly different (*p* > 0.05).

**Table 8:** Rancimat Induction Period (IP) for 0 Days and 3 Days of Storage at 65 °C.

Sample	IP (h) for 0 Days	IP (h) for 3 Days
MH	(5.88 ± 0.31) ^d^	(3.89 ± 0.08) ^d^
MHO-10%	(1.97 ± 0.11) ^e^	(1.42 ± 0.08) ^e^
MHO-15%	(1.90 ± 0.03) ^e,f^	(1.42 ± 0.20) ^e^
MHO-30%	(1.73 ± 0.04) ^e,f,g^	(1.20 ± 0.07) ^e^
MC-10%	(1.38 ± 0.01) ^e,f,g^	(0.75 ± 0.03) ^f^
MC-15%	(1.27 ± 0.06) ^f,g^	(0.75 ± 0.07) ^f^
MC-30%	(1.20 ± 0.04) ^g^	(0.65 ± 0.08) ^f^
MHO-10%-A	(8.03 ± 0.24) ^c^	(7.62 ± 0.21) ^b^
MHO-15%-A	(7.98 ± 0.20) ^c^	(7.88 ± 0.10) ^b^
MHO-30%-A	(8.07 ± 0.13) ^c^	(8.03 ± 0.06) ^b^
Canola Oil	(8.84 ± 0.21) ^b^	(6.20 ± 0.18) ^c^
High-oleic Oil	(17.36 ± 1.13) ^a^	(13.08 ± 0.62) ^a^

Values with the same letter in the same column are not significantly different (*p* > 0.05).

## Data Availability

The original contributions presented in the study are included in the article; further inquiries can be directed to the corresponding author.

## Abbreviations

The following abbreviations are used in this manuscript:

AOAC	Association of Official Analytical Chemists
AOCS	American Oil Chemists’ Society
BHA	Butylated hydroxyanisole
BHT	Butylated hydroxytoluene
BS	Backscattering
│ΔBS│	Variation in BS between time zero and 3 months of storage
D(3,2), D(4,3)	Sauter and De Brouckere diameters, respectively
EDTA	Ethylenediaminetetraacetic acid
F1, F2, F3	Formulations of Mayonnaise-Type Sauce, 10%, 15%, 30% of oil, respectively
FID	Flame Ionization Detector
GC	Gas chromatography
GG	Guar gum
HDLs	High-density lipoproteins
IP	Induction period
IUPAC	International Union of Pure and Applied Chemistry
LDLs	Low-density lipoproteins
LEY	Lyophilized egg yolk
MC	Formulations of Mayonnaise-Type Sauce, X% (10, 15 or 30) of canola oil
MH	commercial mayonnaise
MHO-X%	Formulations of Mayonnaise-Type Sauce, X% (10, 15 or 30) of sunflower high-oleic oil
MHO-X%-A	Formulations of Mayonnaise-Type Sauce, X% (10, 15 or 30) of sunflower high-oleic oil with antioxidant
O/W	Oil in water emulsion
RBN	National Food Regulation (Reglamento Bromatológico Nacional)
TBHQ	Tertiary butylhydroquinone
τ	Shear stress
τ_0_	Yield stress
K	Consistency index
${\dot{\gamma }}^{n}$	${\dot{\gamma }}^{n}$ is the shear rate
n	Flow behavior index
G’ and G’’	Elastic and viscous modulus, respectively
τ_y_	Yield point
τ_f_	Flow point
LVER	Linear viscoelastic region
δ	Phase angle
L*, a*, b*	Luminosity, red-green and yellow-blue coordinates, respectively
